# Information transmission in action video gaming experts: Inferences from the lateralized readiness potential

**DOI:** 10.3389/fnhum.2022.906123

**Published:** 2022-07-25

**Authors:** Jiaxin Xie, Ruifang Cui, Weiyi Ma, Jingqing Lu, Lin Wang, Shaofei Ying, Dezhong Yao, Diankun Gong, Guojian Yan, Tiejun Liu

**Affiliations:** ^1^MOE Key Lab for Neuroinformation, The Clinical Hospital of Chengdu Brain Science Institute, University of Electronic Science and Technology of China, Chengdu, China; ^2^Center for Information in Medicine, School of Life Sciences and Technology, University of Electronic Science and Technology of China, Chengdu, China; ^3^School of Human Environmental Sciences, University of Arkansas, Fayetteville, AR, United States

**Keywords:** action real-time strategy game, asynchronous pattern, lateralized readiness potentials, information transmission, synchronous pattern

## Abstract

Research showed that action real-time strategy gaming (ARSG) experience is related to cognitive and neural plasticity, including visual selective attention and working memory, executive control, and information processing. This study explored the relationship between ARSG experience and information transmission in the auditory channel. Using an auditory, two-choice, go/no-go task and lateralized readiness potential (LRP) as the index to partial information transmission, this study examined information transmission patterns in ARSG experts and amateurs. Results showed that experts had a higher accuracy rate than amateurs. More importantly, experts had a smaller stimulus-locked LRP component (250 – 450 ms) than amateurs on no-go trials, while the response-locked LRP component (0 – 300 ms) on go trials did not differ between groups. Thus, whereas amateurs used an asynchronous information transmission pattern, experts used a reduced asynchronous information transmission pattern or a synchronous pattern where most of processing occurred prior to response execution – an information transmission pattern that supports rapid, error-free performance. Thus, experts and amateurs may use different information transmission patterns in auditory processing. In addition, the information transmission pattern used by experts is typically observed only after long-term auditory training according to past research. This study supports the relationship between ARSG experience and the development of information processing patterns.

## Introduction

Brain and cognitive plasticity (Loevden et al., 2010) occurs when individuals are frequently exposed to cognitively demanding tasks (Dale et al., 2020). Action video gaming (AVG) – an important, new medium of entertainment – requires players to pay close attention to peripheral stimuli while monitoring multiple complex visual stimuli simultaneously under time pressure, thus demanding for a high cognitive load for information processing (Green and Bavelier, 2012), attention (Green and Bavelier, 2003), visual memory (Blacker et al., 2014), and spatial cognition (Green and Bavelier, 2007). Action real-time strategy gaming (ARSG – e.g., League of Legends [LOL]) – an emerging subgenre of AVG – contains (a) “action” components that require sensorimotor skills (e.g., hand-eye coordination, visual attention, and visuospatial cognition) and (b) “strategy” components that require decision making and team collaboration, just like the traditional team sports (e.g., basketball) (Gong et al., 2019a). Thus, ARSG can provide new insights into learning-related cognitive and neural plasticity.

The information transmission pattern across processing stages (i.e., sensory processing, stimulus identification and response selection, preparation and execution stages) has been explored through the transmission speed of various attributes contained by a stimulus (Miller and Hackley, 1992). Even a simple sound stimulus (e.g., a pure tone) contains multiple attributes such as pitch, intensity, and duration. Under certain circumstances, certain attributes can be transmitted to the next stage before other attributes are processed – referred to as partial information transmission (Miller and Hackley, 1992). Event-related potential (ERP) research has examined this effect using both visual and auditory information as the partial information to be transmitted (Osman et al., 1992; Gong et al., 2012b; Lu et al., 2014). There are two competing theories of information transmission pattern. One theory proposes that information transmission occurs in a synchronous pattern, where information processing is completed at each stage before progressing to the next (Sternberg, 1969), resulting in longer response time but higher accuracy by maintaining temporal congruence (Ho et al., 2012). The other theory suggests an asynchronous transmission pattern, where information is processed simultaneously in different stages (Miller and Hackley, 1992; Smulders et al., 1995), thus resulting in shorter response time but lower accuracy due to lack of temporal congruence between stimulus attributes (Ho et al., 2012).

**FIGURE 1:**
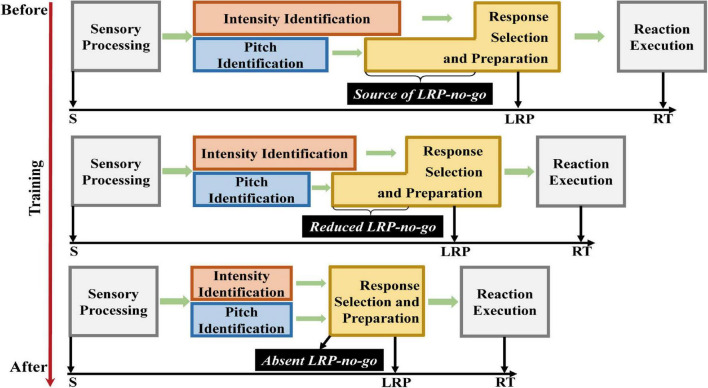
The model showing how training reduces processing times for intensity and pitch until they are comparable and the S-LRP disappears, indicating the development from an asynchronous pattern to a synchronous one. S: Stimulus onset; RT: Reaction time. This Figure is adapted from Miller’s Figure 1 in 1992 (Miller and Hackley, 1992) and Gong’s Figure 5 (Gong et al., 2013).

An interventional study conducted by Gong et al. (2013) examined the effect of a daily 2-h auditory training program that lasted one month on the development of transmission pattern in the auditory channel (Figure 1). Gong et al. explored partial information transmission using pure tones where pitch was more discriminable than intensity. Prior to the training program, pitch was processed faster and transmitted to the response selection and preparation stage before intensity was fully processed, thus leading to the large amplitude of stimulus-locked lateralized readiness potential (S-LRP) on no-go trials which indicates an asynchronous transmission pattern. As the training program progressed, the processing of intensity became faster and the amplitude of S-LRP decreased on no-go trials, indicating the emergence of a reduced asynchronous pattern. Then, as a rapid, error-free performance gradually occurs through learning, the processing of pitch and intensity becomes temporally comparable – as indicated by the disappearance of S-LRP on no-go trials – suggesting that the asynchronous pattern observed prior to training eventually became an error-free synchronous pattern. Thus, the two transmission patterns can be observed in one individual, thus reconciling the two competing theories (Gong et al., 2013). Furthermore, Gong et al. (2013) proposed that the development of information transmission patterns reflects the process of expertise acquisition. Supporting this proposition is the finding that holistic processing can be recruited during the acquisition of expertise in object recognition, and that the level of this recruitment is related to the amount of expertise (Gauthier and Tarr, 2002; Curby et al., 2009; Richler et al., 2011; Chua and Gauthier, 2020). The same logic could hold in auditory processing: the recruitment of holistic processing may be related to the improvement of perceptual discrimination, which can diminish the difference between the processing of pitch and that of intensity, thus reducing the S-LRP component. Furthermore, rapid motor-response preparation was observed after a 5-day visual search practice, as indicated by a significantly earlier S-LRP onset latency after practice (Clark et al., 2015).

Over the past few decades, most of research has shown that AVG experience is related to cognitive and brain plasticity in visual channel (Bediou et al., 2018; Bavelier and Green, 2019), offering a potential pathway for inducing cognitive and brain plasticity. Thus, it is possible that extensive ARSG experience can induce a reduction in the asynchronous pattern and/or the occurrence of an error-free synchronous pattern, just like traditional cognitive and sensorimotor training (Gong et al., 2013; Bavelier and Green, 2019). Supporting this possibility is the fact that ARSG requires players to process a large amount of complex stimuli (e.g., audio-visual stimuli) under time pressure, which can improve their information transmission ability (Lu et al., 2014). Indeed, ARSG experience is associated with cognitive enhancement, such as cognitive flexibility (Glass et al., 2013), executive control (Li et al., 2020), and fluid intelligence (Kokkinakis et al., 2017). Long-term ARSG/real-time strategy video game (e.g., LOL) experience is related to the enhancement in the functional integration of insular subregions and the pertinent networks therein (Gong et al., 2015), that between salience and central executive networks (Gong et al., 2016), and the alterations along axons that link structures of the occipito-parietal loop related to spatial and visual processing (Kowalczyk et al., 2018). ARSG experience is also related to improvement in the spatial (Qiu et al., 2018) and temporal component (Gan et al., 2020) of visual selective attention and working memory (Yao et al., 2020) as indicated by ERP measures. Moreover, electrophysiological (EEG) network characteristics reveal a more active information processing during ARSG than low cognitive-load states (Gong et al., 2019a). All these cognitive functions are closely related to components essential for information transmission, such as attention (Stelmach and Herdman, 1991; Saalmann et al., 2012), work memory (Xia et al., 2019; Zhang et al., 2020), and perceptual discriminability (Gong et al., 2012b), thus opening up the possibility that ARSG experience is related to improved information transmission.

This study explores the effect of the ARSG experience on information transmission in auditory channel by determining whether ARSG (e.g., LOL) experts have a superior information transmission pattern compared with ARSG (e.g., LOL) amateurs. This study differs from past research in two ways. First, while past research mostly focused on the effect of AVG experience on *visual* development (Bediou et al., 2018; Bavelier and Green, 2019), this study examined the relationship between AVG experience and *auditory* development (also read Donohue et al., 2010; Zhang et al., 2017; Cui et al., 2021). The in-game sound of ARSG (e.g., LOL) is essential for successful gaming experience. For example, the sound of fixed skills assists players in predicting enemies’ trajectory of motion (e.g., the sound of Ash’s R skill), while the sound of additional skills enables players to determine whether certain skills are released to save teammates or kill enemies (e.g., the sound of the kindling skill), and the sound of non-player controlled characters assists players in inferring what is outside their field of vision (e.g., the sound made when monsters are attacked or killed). Thus, LOL experience may be related to improved auditory abilities.

Second, to our knowledge, this study is the first to examine the relationship between individuals’ AVG experience and their *information transmission pattern* in auditory channel. Participants were administered a two-choice auditory go/no-go reaction-time task, which used pure tones whose pitch was more discriminable than intensity. According to past research (Gong et al., 2013), this study defined partial information transmission as the transmission of pitch to the next stage before intensity. If the asynchronous pattern decreased and/or the error-free synchronous pattern started to occur in LOL experts, we would predict that when compared with amateurs, LOL experts should have a higher accuracy rate, fast reaction time, and a smaller difference in their perceptual sensitivity (*d*′) between pitch and intensity. In addition, this study used the lateralized readiness potential (LRP) – a measure that offers a higher temporal resolution than behavioral index (Luck, 2014) – to examine partial information transmission. LRP negative-wave can reflect asymmetrical cortical activation as indicated by the difference between the contra- and ipsilateral amplitudes related to responding hands when subjects are informed which hand to use to respond (Gratton et al., 1988; Coles, 1989). S-LRP is associated with response selection and preparation, while response-locked LRP (LRP-R) may reflect the response organization and execution of the motor response (Coles, 1989; De Sanctis and Sommer, 2009). If LOL experts have a reduced asynchronous pattern and/or an emerging error-free synchronous pattern, the mean amplitudes of S-LRP should be smaller in experts than in amateurs on no-go trials, while the mean amplitudes of LRP-R should not significantly differ between groups (Coles, 1989), thus indicating that in experts, most processing has occurred prior to response execution (Gong et al., 2013).

## Materials and methods

### Participants

This study used the recruitment procedure established by previous research (Qiu et al., 2018; Gong et al., 2019b; Gan et al., 2020; Yao et al., 2020). All participants in this study were college students at the University of Electronic Science and Technology of China (UESTC) who responded to the recruitment flyers posted on campus or on Internet forums hosted by UESTC. Prior to the experiment, participants provided their demographic information including their age, gender, handedness, vision, audition, and history of mental and neurological diseases. All participants were right-handed, had normal audition, normal or corrected-to-normal vision, and no history of mental or neurological diseases. Participants also reported (1) their experience of LOL – the ARSG game used in this study – over the past two years and their Expertise Gaming Ranking, (2) their LOL ID that was used to validate their self-reported gaming experience and expertise, and (3) their gaming experience of other game genres over the immediate recent two years, which was used to ensure that LOL was the primary game genre for all participants, including both experts and amateurs recruited in this study (Qiu et al., 2018; Gong et al., 2019b; Gan et al., 2020; Yao et al., 2020).

League of legends experts and amateurs were defined according to both time- and skill-based criteria established in previous research (Qiu et al., 2018; Gong et al., 2019b; Gan et al., 2020; Yao et al., 2020). The experts had at least 2 years of LOL gaming experience and were regarded as LOL masters based on their expertise gaming ranking (the top 7% of players), an objective, generally used tool for measuring the relative gaming skill of LOL players. The amateurs had less than 0.5 years of LOL gaming experience and were regarded as amateurs based on their expertise gaming rankings (the lowest 29.92–45.11% of players). The participants were 22 male LOL amateurs (*M* = 21.42, *SD* = 3.20) and 22 male LOL experts (*M* = 20.08, *SD* = 2.93). The sample size was determined using G*Power 3.1.9 with an effect size of 0.25, a power of 0.80, and an alpha of 0.05 (Faul et al., 2007). To minimize participant bias, the participants were not informed of their group membership or the aim of the study.

### Stimuli and apparatus

Table 1 shows the types of stimuli used in this study. Participants were seated approximately 1.1 m in front of a computer screen in a dimly lit, soundproofed, and electrically shielded room. Following the procedure used by Gong et al. (2013), four pure tone stimuli (1,000 Hz vs. 2,000 Hz; 55 dB vs. 59 dB) were created using Adobe Audition 3.0 and verified using Praat. Pure tones with a duration of 375 ms were binaurally presented via Sennheiser headphones. The sound pressure level was monitored through a digital sound level meter.

**TABLE 1:** The types of stimuli used in this study.

Pitch	Intensity	Left/Right-hand	Go/No-go response	Trials
1000 Hz	55 dB	Left-hand	No-go	160
1000 Hz	59 dB	Left-hand	Go	320
2000 Hz	55 dB	Right-hand	No-go	160
2000 Hz	59 dB	Right-hand	Go	320

### Design and procedure

An experiment consisted of eight blocks, each consisting of 120 trials which were presented in random order. On each trial, participants heard either a louder (59 dB) or softer (55 dB) high-pitch (1,000 Hz) or low-pitch (2,000 Hz) pure tone. The pitch of the tone indicated which hand to use, while its intensity indicated whether a response should be performed (“go” or “no-go”). The stimulus response assignments remained constant within a participant, and were counter-balanced across participants. For each participant, left-hand and right-hand responses were equally frequent, while the likelihood of the go and no-go trials was 0.67 and 0.33, respectively.

Each trial started with a cross (+) presented in the center of a black screen. The participants were asked to focus on the cross throughout an experiment. Approximately 500 ms before the imperative stimulus, the cross flashed to prompt the participants of the upcoming pure tone (duration = 375 ms). On the go trials, participants were asked to respond as quickly and accurately as possible by pressing three keys (the C, Z, and X keys on a standard keyboard for left-hand responses, and the M, period keys, and comma for right-hand responses) with the index, ring, and middle fingers. The C and M keys were “correct” responses. This design was used because the robustness of the LRP component is related to the complexity of action required by the task (Hackley and Miller, 1995). On the no-go trials, participants were asked to avoid making any overt finger response. After a random inter-trial interval of 1,250-1,500 ms, the next trial began. An experiment started with a practice phase, during which feedback (1,500 ms) concerning correctness and speed of responses was provided immediately after each response or 2 s later when a response should be prohibited. After 30 practice trials, the main experiment started. Participants were not provided with feedback in the main experiment.

### Electrophysiological recording and preprocessing

The behavioral data and EEG signals were recorded synchronously. The EEG data were collected using an electrode cap with 32 Ag-AgCl electrodes based on an international 10-20 system. Following the procedure used in previous studies (Gan et al., 2020; Yao et al., 2020), the EEG signals were recorded using an amplifier (BioAmp-eeg32) designed by the Key Laboratory of the Ministry of NeuroInformation Education of the UESTC, and was digitized with a 1,000-Hz sampling rate. All signals were referenced online with linked earlobes and filtered online with 0.05-100 Hz. To monitor eye movement artifacts, vertical electrooculograms (EOGs) and horizontal EOGs were recorded. The impedances of all electrodes were kept below 5 KΩ.

The offline EEG data were analyzed using EEGLAB (Delorme and Makeig, 2004) and ERPLAB (Lopez-Calderon and Luck, 2014) toolboxes in MATLAB 2013b (MathWorks, Natick, NA). Raw data were first re-referenced to “infinity” zero provided offline by the reference electrode standardization technique (REST) (Yao, 2001). The data were then filtered using IIR-Butterworth non-causal filters with half-power cutoffs of 0.10 Hz and 30 Hz (roll-off = 12 dB/oct) and 2 order. A 180-order, 50-Hz Parks-McClellan Notch Filter was used to avoid 50-Hz-frequency interference. Next, independent component analysis (ICA) was performed for eye movement correction. For the S-LRP component, the preprocessed EEG data were segmented into 1,000 ms epochs that were time-locked to stimulus onset and included a 200 ms prestimulus baseline. For the LRP-R component, the preprocessed EEG data were segmented into 1,200 ms epochs that were time-locked to the response onset and included 200-ms postresponse data, and the baseline ranged from −1000 to −800 ms before response onset. Noise was removed using the sliding window with a 200 ms window width, 100 ms window step, and ± 100 μV threshold (Luck, 2014). In addition, for go trials, the correct trials were defined as the trials of correct hits. Specifically, for go trials that required left-hand (or right-hand) responses, the correct trials were the trials where left-hand (or right-hand) responses were made. For no-go trials, the correct trials were the trials of correct rejections. Only “correct” trials were included for further analysis.

### Data analysis

The accuracy rate and reaction time on “correct” trials were analyzed. For go trials, the accuracy rate was defined as the correct hit rates; for no-go trials, the accuracy rate was defined as the correct rejection rates. For go trials, reaction time was defined as the time interval between the pure tone onset and the participants’ first key response, a C (left-hand response) or M key response (right-hand response). The trials of left- and right-hand response were classified and averaged to ensure statistical power. For the accuracy rate, a 2 (group: experts, amateurs) × 2 (response: go, no-go) two-way ANOVA analysis was performed. For the reaction time data, an independent-samples *t*-test compared reaction time between LOL experts and amateurs on go trials.

In addition, *d’* was calculated according to the hit and false alarm rates (Gong et al., 2012a,^2013^). To calculate *d’*, one stimulus dimension was arbitrarily defined as the signal, while the other was defined as the noise. In this study, the intensity related to go responses was the signal and that related to no-go responses was the noise; the pitch related to left-hand responses was the signal and that related to right-hand responses was the noise. Then, the hit and false alarm rates for the pitch and intensity were calculated respectively (Miller and Hackley, 1992). For pitch, a hit was defined as a left-hand response to a stimulus requiring a left-hand response, regardless of whether the intensity of the tone indicated a go or a no-go response; a false alarm was defined as a left-hand response to the other pitch. For intensity, a hit was defined as a go response to a go stimulus, regardless of whether the pitch of the tone indicated a left-hand or a right-hand response; a false alarm was defined as a go response to a no-go stimulus. Then, *d’* was calculated. For the *d’* data, a 2 (group: experts vs. amateurs) × 2 (stimulus: pitch vs. intensity) two-way ANOVA was performed.

According to group-average topographic maps and waveforms, the mean amplitudes of N1 and P2 components were evaluated during 90 – 130 ms and 160 – 200 ms at FCz electrode after the stimulus onset, respectively. Since this study focused on the LRP component, C3 and C4 electrodes were selected for further analysis (Gong et al., 2012a,b, 2013; Lu et al., 2014). The LRP was extracted based on the difference between the contra- and ipsilateral electrodes over the primary motor cortex time-locked to stimulus onset (S-LRP) or response onset (LRP-R) and the contra- and ipsilateral electrodes connected to the responding hand (Gong et al., 2013; Uccelli et al., 2020). Specifically, the LRP was calculated by subtracting the ipsilateral amplitude from the contralateral amplitude (i.e., C3–C4 and C4–C3 for the right- and left-hand responses, respectively) for the left-hand go trials (C4-C3), left-hand no-go trials (C4-C3), right-hand go trials (C3-F4), and right-hand no-go trials (C3-C4). Then, according to whether the key was pressed or not (go/no-go), the above four conditions were merged into two conditions: go trials and no-go trials. Finally, based on previous studies and group-level waveforms (Takacs et al., 2020; Walk et al., 2020), the mean amplitudes of S-LRP were evaluated from 250 to 450 ms from the stimulus presentation, and the LRP-R mean amplitudes were evaluated from −300 to 0 ms to the responses, where the mean amplitudes of LRP-R were measured every 50 ms. For the LRP-S component, a 2 (group: experts vs. amateurs) × 2 (response: go vs. no-go) two-way ANOVA was performed. For the R-LRP component, independent-samples *t*-tests compared the mean amplitudes between experts and amateurs on the go trials within each time window.

## Results

### Behavioral results

Reaction time on the “correct” trials did not differ between right- (experts: 789.80 ms; amateurs: 846.85 ms) and left-hand responses (experts: 799.74 ms; amateurs: 848.62 ms) in either experts (*t* = 0.54, *p* = 0.59) or amateurs (*t* = 0.08, *p* = 0.93), suggesting that no confusion occurred in participants’ key pressing responses when they were asked to press three keys rather than one. Then, the trials of left-hand and right-hand responses were classified and averaged for further analysis. Within each participant, we calculated an average reaction time on “correct” go trials. However, an independent-samples *t*-test found that reaction time did *not* significantly differ between experts (*M* = 794.77 ms, *SE* = 22.71 ms) and amateurs (*M* = 847.73 ms, *SE* = 28.27 ms; *t* = 1.46, *p* = 0.15).

For the accuracy rate, a two-way ANOVA showed that the main effect of group (*F* [1, 42] = 5.50, *p* = 0.02, η^2^*_*p*_* = 0.12) was significant, but neither the group × response interaction nor the main effect of response approached significance (*p’s* > 0.05). These findings suggested that (a) the accuracy rate was higher in experts (*M* = 89.18%, *SE* = 0.94%) than in amateurs (*M* = 84.22%, *SE* = 1.90%) – a finding that applied to both types of responses (i.e., go, no-go), and (b) the accuracy rate did not differ between go and no-go responses. Then, we performed independent-samples t-tests to compare the accuracy rate of LOL experts and amateurs on go trials and no-go trials, respectively. On go trials, experts (*M* = 91.32%, *SE* = 1.07%) had higher accuracy rate than amateurs (*M* = 81.72%, *SE* = 2.50%), *t* = 3.49, *p* = 0.001, Cohen’s *d* = 1.05. However, on no-go trials, no significant between-group differences emerged (experts: *M* = 87.06%, *SE* = 1.84%; amateurs: *M* = 86.70%, *SE* = 2.95%; *t* = 0.12, *p* = 0.90).

Table 2 shows the percentage of different stimuli for each type of response. First, we performed independent-samples *t*-test to compare the false alarm rates between experts and amateurs for pitch and intensity, respectively. No significant difference was found between the two groups for ether pitch (experts: *M* = 0.53%, *SE* = 0.14%; amateurs: *M* = 0.87%, *SE* = 0.36%; *t* = 0.90, *p* = 0.37) or intensity (experts: *M* = 12.94%, *SE* = 1.83%; amateurs: *M* = 13.30%, *SE* = 2.94%; *t* = 0.10, *p* = 0.92). Then for the *d’* data, a two-way ANOVA showed that the main effects of group (*F* [1, 42] = 3.55, *p* = 0.07, η^2^*_*p*_* = 0.08) and stimulus (*F* [1, 42] = 25.58, *p* < 0.001, η^2^*_*p*_* = 0.38) were marginally significant and significant, respectively, but the group × stimulus interaction was not significant (*F* [1, 42] = 0.92, *p* = 0.34). These findings suggested that *d’* was marginally higher in experts (*M* = 2.91, *SE* = 0.10) than in amateurs (*M* = 2.65, *SE* = 0.10) – a finding that applied to both types of stimuli (i.e., pitch, intensity). Furthermore, *d’* significantly decreased from pitch (*M* = 3.01, *SE* = 0.07) to intensity (*M* = 2.55, *SE* = 0.10) – a finding that applied to both groups of participants. An independent-samples *t*-test found that the degree of this decrease (pitch *d’* minus intensity *d’*) did not differ between experts (*M* = 0.37, *SE* = 0.14) and amateurs (*M* = 0.54, *SE* = 0.12); *t* = 0.96, *p* = 0.34.

**TABLE 2:** Percentage of different stimulus in each type of response.

Groups	Responses	Stimulus			
		
		Left go (%)	Left no-go (%)	Right go (%)	Right no-go (%)
Amateurs	Left go	81.60	14.27	0.99	0.51
	Right go	0.89	0.34	81.84	11.49
	No-go	17.51	85.39	17.17	88.00
Experts	Left go	91.51	14.22	0.65	0.20
	Right go	0.67	0.12	91.12	11.35
	No-go	7.82	85.66	8.23	88.45

### Event-related potential results

**FIGURE 2:**
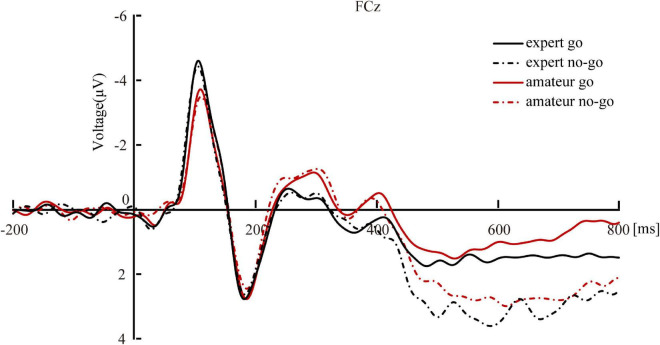
N1 and P2 mean amplitudes to the go and no-go trials for LOL experts and amateurs. The zero on the x-axis indicates the onset of stimulus. The electrodes selected were FCz.

**FIGURE 3:**
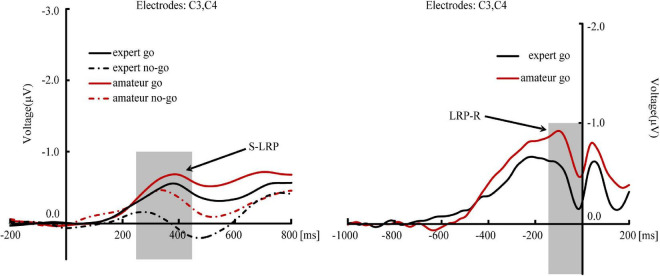
Event-related potential responses to the go and no-go trials for LOL experts and amateurs. The left figure shows the event-related potential responses on the go and no-go trials that are stimulus-locked. The right figure shows the event-related potential responses to the go trials that are response-locked. The zero on the x-axis indicates the onset of stimulus in the left and the onset of responses in the right. The electrodes selected were C3 and C4. The shadow box indicates the time window of the LRP component. S-LRP indicates stimulus-locked lateralized readiness potential; LRP-R indicates response-locked lateralized readiness potential.

Separate independent samples *t* tests compared the mean amplitudes of N1 and P2 between experts and amateurs. Results showed no significant between-group differences for either N1 or P2 (*p’s* > 0.18; Figure 2). Figure 3 shows the event-related potentials of the go and no-go trials. The left figure shows the event-related potentials time-locked to the stimulus for the go and no-go trials. A 2 (group: experts vs. amateurs) × 2 (response: go vs. no-go) two-way ANOVA on the mean amplitudes during 250-450-ms time window found that neither the main effect of group nor the group × response interaction was significant (*p’s* > 0.09), but the main effect of response was significant (*F* [1, 42] = 22.01, *p* < 0.001, η^2^*_*p*_* = 0.34). These findings suggested that (a) the mean amplitudes did not differ between groups, (b) the mean amplitudes of S-LRP were greater with go responses (*M* = −0.56 μV, *SE* = 0.09 μV) than with no-go responses (*M* = −0.22 μV, *SE* = 0.07 μV) – a finding that applied to both groups of participants. Then, *post hoc* independent-samples *t*-tests examined the between-group differences in the S-LRP component on no-go and go trials, respectively. On no-go trials, S-LRP amplitudes were smaller in experts (*M* = −0.04 μV, *SE* = 0.11 μV) than in amateurs (*M* = -0.41 μV, *SE* = 0.10 μV; *t* = −2.47, *p* = 0.02, Cohen’s *d* = 0.75). However, on go trials, S-LRP amplitudes did not differ between experts (*M* = −0.49 μV, *SE* = 0.14 μV) and amateurs (*M* = −0.63 μV, *SE* = 0.11 μV; *t* = 0.44, *p* = 0.32).

To determine whether the S-LRP existed on no-go trials during 250-450 ms, *t*-tests compared the mean amplitudes during 250-450 ms against those during the baseline interval (i.e., 200 ms before stimulus) on no-go trials within experts and amateurs, respectively. The mean amplitudes between the two time windows was significantly different in amateurs (*t* = 4.19, *p* < 0.001, Cohen’s *d* = 1.15) but not in experts (*t* = 0.39, *p* = 0.70). In addition, for go trials, the mean amplitudes between the two time windows was significantly different in both amateurs (*t* = 5.54, *p* < 0.001, Cohen’s *d* = 1.49) and experts (*t* = 3.45, *p* = 0.002, Cohen’s *d* = 0.86). Moreover, we also compared the mean amplitudes between go and no-go trials in the 150-200-ms and 200-250-ms time windows within experts and amateurs, respectively. We found that prior to the onset of partial information transmission, electrophysiological activity to pitch and intensity did not differ in either experts or amateurs in these time windows (*p’s* > 0.28).

The right figure of Figure 3 shows the event-related potentials time-locked to responses for go trials. Table 3 shows the results of the LRP-R component during 0-300 ms before responses, where the mean amplitudes of LRP-R were measured every 50 ms. Separate independent-samples *t*-tests showed that the mean amplitudes were marginally smaller in experts than in amateurs in the 0-50-ms and 50-100-ms time windows, but did not differ between groups in other time windows.

**TABLE 3:** The results of LRP-R component during 0-300 ms before responses.

Time windows	Groups	Mean (SE) μV	*T*	*P*	Cohen’s *d*
0-50 ms	Amateurs	−0.40 (0.15)	1.90	0.07	0.57
	Experts	−0.02 (0.14)			
50-100 ms	Amateurs	−0.93 (0.14)	1.79	0.08	0.54
	Experts	−0.57 (0.15)			
100-150 ms	Amateurs	−0.91 (0.14)	1.39	0.17	0.42
	Experts	−0.63 (0.14)			
150-200 ms	Amateurs	−0.83 (0.16)	0.83	0.41	0.25
	Experts	−0.66 (0.14)			
200-250 ms	Amateurs	−0.84 (0.16)	0.70	0.49	0.21
	Experts	−0.69 (0.14)			
250-300 ms	Amateurs	−0.72 (0.14)	0.59	0.56	0.18
	Experts	−0.60 (0.14)			

## Discussion

Using both behavioral and LRP measures, this study examined information transmission in LOL experts and amateurs under an auditory, two-choice go/no-go paradigm. Results showed that experts had a higher accuracy rate than amateurs. Furthermore, S-LRP amplitudes were smaller in experts than in amateurs on no-go trials, but LRP-R amplitudes did not differ between groups on go trials. Thus, in LOL experts, most of processing has occurred prior to response execution, which indicated that compared with amateurs, LOL experts might have a reduced asynchronous pattern or an emerging synchronous pattern (Gong et al., 2013).

League of legends experts tended to outperform amateurs in the current task. This is supported by the findings that (a) the accuracy rate was higher in experts than in amateurs, which is consistent with past findings that ARSG experience was associated with improved cognitive functions, including cognitive flexibility (Glass et al., 2013), executive control (Li et al., 2020), fluid intelligence (Kokkinakis et al., 2017), selective attention (Qiu et al., 2018; Gan et al., 2020), visual working memory (Yao et al., 2020), and information processing (Gong et al., 2019a), and (b) *d’* was higher in experts than amateurs – a finding that applied to both pitch and intensity processing. Moreover, the results in accuracy rate also suggest experts pressed more correct response keys on go trials than amateurs, while the correct rejection rate on no-go trials did not differ between groups. However, reaction time did not significantly differ between groups. Perhaps, under the current experimental paradigm, reaction time was a less sensitive indicator to one’s behavioral performance than accuracy. Thus, between-group differences in reaction time may be less observable than those in accuracy in this study. Meanwhile, our finding also implied the difference between experts and amateurs is not in false alarm rates that reflects proactive inhibition controls.

This study found that S-LRP amplitudes were smaller in experts than in amateurs on no-go trials, but LRP-R amplitudes did not differ between groups on go trials. This finding suggested that in LOL experts, most of processing occurred prior to response execution. Thus, compared with amateurs, LOL experts might have a reduced asynchronous pattern or an emerging synchronous pattern (Gong et al., 2013). The LRP component may reflect the response selection, preparation, and executions of the motor cortex (Coles, 1989). The S-LRP can be associated with premotor processing, such as response selection and preparation (Coles, 1989), while the LRP-R reflects motor organization and execution (Coles, 1989; Masaki et al., 2004; De Sanctis and Sommer, 2009). In addition, S-LRP component existed on no-go trials during 250-450 ms in amateurs, suggesting amateurs did response preparation (Gong et al., 2012b). Then, the difference between go and no-go trials suggested the response preparation was not based on the whole information (i.e., the two attributes) but on the partial information of the stimulus (i.e., pitch) (Gong et al., 2012b).

The model proposed by Gong et al. (2013) explains how learning induces plasticity in information processing and transmission patterns (Gong et al., 2013; Figure 1). Long-term learning can decrease S-LRP amplitudes on no-go trials. Thus, learning can transform the information transmission pattern from an asynchronous pattern to a reduced asynchronous pattern and eventually to a rapid, error-free synchronous pattern. This may reflect the process of expertise acquisition. The experience of playing ARSG – a popular platform of intensive learning in the modern society – is related to enhanced cognitive flexibility (Glass et al., 2013), executive control (Li et al., 2020), intelligence (Kokkinakis et al., 2017), attention (Qiu et al., 2018; Gan et al., 2020), visual work memory (Yao et al., 2020), information processing (Gong et al., 2019a), and related neural mechanisms (Gong et al., 2015, 2016). These cognitive functions are closely related to information transmission. For example, clusters of cortical neurons can synchronize their activity to preferentially transmit information with attentional priorities (Saalmann et al., 2012). Directed attention may affect the speed of information transmission in the visual system (Stelmach and Herdman, 1991). Direct ventral hippocampus-medial prefrontal cortex information transmission may affect spatial working memory in rats (Xia et al., 2019). Furthermore, correct storage of work memory information can increase the network connection and efficiency of information transmission (Zhang et al., 2020). These findings support the link between long-term ARSG experience and an advanced information transmission pattern.

The difference of mean amplitudes between 250-450-ms after stimulus and the 200-ms baseline before stimulus in amateurs indicated that the amateurs used an asynchronous transformation pattern. Furthermore, partial information transmission was not observed in the LOL experts, suggesting the experts had a reduced asynchronous transformation pattern or even an emerging synchronous pattern. Meanwhile, the LRP-R amplitudes did not significantly differ between groups, suggesting that there was no significant between-group difference in response execution, and that the difference in S-LRP component on no-go trials between the two groups was not simply due to reduced movement complexity (Hackley and Miller, 1995). Thus, the current study suggests that LOL experts and amateurs may use different information transmission patterns in auditory processing. In addition, the pattern used by experts is associated with more accurate and efficient information processing – an information transmission pattern that is typically observable only after long-term auditory training (Gong et al., 2013).

This study found that LOL experts used a different auditory information transformation pattern than amateurs. The current findings are consistent with past findings that are mostly focused on the effect of AVG experience on the development of visual abilities (Bediou et al., 2018; Bavelier and Green, 2019). Why is LOL experience related to the development of auditory information transformation? There are two explanations. First, it may be related to the “learning to learn” hypothesis that AVG experts are generally good at learning task-relevant information (Bavelier et al., 2012). This explanation predicts that the benefit of AVG experience is observable when other cognitive abilities or domains are evaluated. However, owing to the correlational nature of this study, the current finding does not allow us to verify a further prediction made by the “learning to learn” hypothesis on *how* AVG experience facilitates cognitive development. Bavelier et al. (2012) also proposed that AVG facilitates cognitive development because AVG teaches players quick learning skills, thereby improving their capability to quickly learn to perform new tasks. Future interventional research should examine how AVG experience facilitates one’s general quick learning ability. Second, LOL gaming is a multi-sensory experience where auditory processing is also important, because sounds can be used to complete various tasks, such as communication with teammates, identification of the nature and source of appearing stimuli, and examination and understanding of the ever-changing battlefield situation. This explanation is consistent with past findings that AVG/video game experience was related to improved auditory cognition (Green et al., 2010; Zhang et al., 2017) and multisensory perception (Donohue et al., 2010).

In addition, this study found that LOL experts had improved intensity transmission but not pitch transmission when compared with amateurs. It is important to note that this finding by no means indicates that AVG experience cannot improve pitch transmission or intensity transmission is more improvable than pitch transmission. The current finding should be related to the experimental design used in this study. This study used pure tones whose pitch was more discriminable (1,000 Hz vs. 2,000 Hz) than intensity (55 dB vs. 59 dB). This is an established experiment design used to study partial information (Gong et al., 2012b), based on the logic that the attribute with high perceptual discriminability (pitch in this study) may be transmitted earlier than the attribute with low perceptual discriminability (intensity in this study). LOL experts may have improved transmission of the information that is lowly discriminable and therefore difficult to perceive, consistent with learning theory (Wenger et al., 2017; Lindenberger and Lövdén, 2019). The discrimination of pitch (1,000 Hz vs. 2,000 Hz) should be exceptionally easy, which may leave one’s pitch discrimination performance irrelevant to their learning experience (AVG experience in this study). We would predict that LOL experts should have improved pitch transmission rather than intensity transmission if we use pure tones whose intensity was more discriminable (e.g., 30 dB vs. 80 dB) than pitch (1,000 Hz vs. 1100 Hz) when compared with amateurs. This prediction is supported by past findings that partial information transmission is modulated by attribute’s perceptual discriminability regardless of its relation to the category identity of a stimulus (e.g., shape and pitch) (Kopp and Wessel, 2010; Gong et al., 2012b). Nevertheless, future research should examine this prediction.

Nevertheless, the correlational nature of this study precludes drawing causal conclusions. It is impossible to rule out the possibility that there are pre-existing systematic differences between the experts and amateurs recruited in this study. To address this issue, researchers should conduct a longitudinal, interventional study, where participants are randomly assigned to receive (or not to receive) AVG training. This design can address whether intentional training of ARSG can improve auditory information transformation. Furthermore, it is also important to examine how various factors (e.g., amount of gaming time, game content) affects the balance of the significance of ARSG use between improved information processing and negative consequences (Bediou et al., 2018; Dale et al., 2020). For example, certain game elements (e.g., virtual reward system, including caparison, power-ups, storyline missions, and daily login gifts) may influence players’ gaming behaviors and habits, thereby preventing cognitive development. Future research should examine how various game elements contribute to cognitive improvement, thus laying the theoretical framework for developing the next-generation AVG-based tool to facilitate cognitive development.

## Conclusion

This study found that LOL experts had higher accuracy than amateurs in an auditory perception task. Moreover, in the experts, most of processing has occurred prior to response execution, suggesting LOL experts and amateurs used different information transmission patterns and that the pattern used by experts is associated with more accurate and efficient information transmission. Thus, long-term ARSG experience was related to the development of information transmission.

## Data availability statement

The raw data supporting the conclusions of this article will be made available by the authors, without undue reservation.

## Ethics statement

The studies involving human participants were reviewed and approved by the Ethics Committee of the University of Electronic Science and Technology of China. The patients/participants provided their written informed consent to participate in this study.

## Author contributions

JX, DY, DG, GY, and TL contributed to conception and design of the study. JX, RC, JL, LW, and SY collected the data. JX, WM, JL, LW, and SY performed the statistical analysis. JX, RC, and WM wrote the original draft of the manuscript. All authors contributed to manuscript revision, read, and approved the submitted version.

## References

[B1] Bavelier D. Green C. S. (2019). Enhancing attentional control: lessons from action video games. *Neuron* 104 147–163. 10.1016/j.neuron.2019.09.031 31600511

[B2] Bavelier D. Green C. S. Pouget A. Schrater P. (2012). Brain plasticity through the life span: learning to learn and action video games. *Annu. Rev. Neurosci.* 35 391–416. 10.1146/annurev-neuro-060909-152832 22715883

[B3] Bediou B. Adams D. M. Mayer R. E. Tipton E. Green C. S. Bavelier D. (2018). Meta-analysis of action video game impact on perceptual, attentional, and cognitive skills. *Psychol. Bull.* 144 77–110. 10.1037/bul0000130 29172564

[B4] Blacker K. J. Curby K. M. Klobusicky E. Chein J. M. (2014). Effects of action video game training on visual working memory. *J. Exp. Psychol. Hum. Percept. Perform.* 40 1992–2004. 10.1037/a0037556 25068696

[B5] Chua K.-W. Gauthier I. (2020). Domain-specific experience determines individual differences in holistic processing. *J. Exp. Psychol. Gen.* 149 31–41. 10.1037/xge0000628 31144835

[B6] Clark K. Appelbaum L. G. van den Berg B. Mitroff S. R. Woldorff M. G. (2015). Improvement in visual search with practice: mapping learning-related changes in neurocognitive stages of processing. *J. Neurosci.* 35 5351–5359. 10.1523/jneurosci.1152-14.2015 25834059 PMC4381005

[B7] Coles M. G. (1989). Modern mind-brain reading: psychophysiology, physiology, and cognition. *Psychophysiology* 26 251–269. 10.1111/j.1469-8986.1989.tb01916.x 2667018

[B8] Cui R. Jiang J. Zeng L. Jiang L. Xia Z. Dong L. (2021). Action video gaming experience related to altered resting-state EEG temporal and spatial complexity. *Front. Hum. Neurosci.* 15:640329. 10.3389/fnhum.2021.640329 34267631 PMC8275975

[B9] Curby K. M. Glazek K. Gauthier I. (2009). A visual short-term memory advantage for objects of expertise. *J. Exp. Psychol. Hum. Percept. Perform.* 35 94–107. 10.1037/0096-1523.35.1.94 19170473 PMC4159943

[B10] Dale G. Joessel A. Bavelier D. Green C. S. (2020). A new look at the cognitive neuroscience of video game play. *Ann. N.Y. Acad. Sci.* 1464 192–203. 10.1111/nyas.14295 31943260

[B11] De Sanctis P. Sommer W. (2009). Information transmission for one-dimensional stimuli: the role of strategies. *Acta Psychol.* 131 12–23. 10.1016/j.actpsy.2009.02.001 19268876

[B12] Delorme A. Makeig S. (2004). EEGLAB: an open source toolbox for analysis of single-trial EEG dynamics including independent component analysis. *J. Neurosci. Methods* 134 9–21. 10.1016/j.jneumeth.2003.10.009 15102499

[B13] Donohue S. E. Woldorff M. G. Mitroff S. R. (2010). Video game players show more precise multisensory temporal processing abilities. *Attent. Percept. Psychophys.* 72 1120–1129. 10.3758/app.72.4.1120 20436205 PMC3314265

[B14] Faul F. Erdfelder E. Lang A.-G. Buchner A. (2007). G*Power 3: a flexible statistical power analysis program for the social, behavioral, and biomedical sciences. *Behav. Res. Methods* 39 175–191. 10.3758/bf03193146 17695343

[B15] Gan X. Yao Y. Liu H. Zong X. Cui R. Qiu N. (2020). Action real-time strategy gaming experience related to increased attentional resources: an attentional blink study. *Front. Hum. Neurosci.* 14:101. 10.3389/fnhum.2020.00101 32341688 PMC7163005

[B16] Gauthier I. Tarr M. J. (2002). Unraveling mechanisms for expert object recognition: bridging brain activity and behavior. *J. Exp. Psychol. Hum. Percept. Perform.* 28 431–446. 10.1037//0096-1523.28.2.431 11999864

[B17] Glass B. D. Maddox W. T. Love B. C. (2013). Real-time strategy game training: emergence of a cognitive flexibility trait. *PLoS One* 8:e70350. 10.1371/journal.pone.0070350 23950921 PMC3737212

[B18] Gong D. He H. Liu D. Ma W. Dong L. Luo C. (2015). Enhanced functional connectivity and increased gray matter volume of insula related to action video game playing. *Sci. Rep.* 5:9763. 10.1038/srep09763 25880157 PMC5381748

[B19] Gong D. He H. Ma W. Liu D. Huang M. Dong L. (2016). Functional integration between salience and central executive networks: a role for action video game experience. *Neural Plast.* 2016:9803165. 10.1155/2016/9803165 26885408 PMC4739029

[B20] Gong D. Hu J. Yao D. (2012a). Partial information can be transmitted in an auditory channel: inferences from lateralized readiness potentials. *Psychophysiology* 49 499–503. 10.1111/j.1469-8986.2011.01325.x 22176682

[B21] Gong D. Ma W. Hu J. Hu Q. Lai Y. Yao D. (2012b). The flexibility of partial information transmission in the auditory channel: the role of perceptual discriminability. *Psychophysiology* 49 1394–1400. 10.1111/j.1469-8986.2012.01452.x 22905969

[B22] Gong D. Li Y. Yan Y. Yao Y. Gao Y. Liu T. (2019a). The high-working load states induced by action real-time strategy gaming: an EEG power spectrum and network study. *Neuropsychologia* 131 42–52. 10.1016/j.neuropsychologia.2019.05.002 31100346

[B23] Gong D. Yao Y. Gan X. Peng Y. Ma W. Yao D. (2019b). A reduction in video gaming time produced a decrease in brain activity. *Front. Hum. Neurosci.* 13:134. 10.3389/fnhum.2019.00134 31057383 PMC6478706

[B24] Gong D. Ma W. Kendrick K. M. Hu Q. Yao D. (2013). How cognitive plasticity resolves the brain’s information processing dilemma. *Sci. Rep.* 3:2860. 10.1038/srep02860 24091591 PMC3790200

[B25] Gratton G. Coles M. G. Sirevaag E. J. Eriksen C. W. Donchin E. (1988). Pre- and poststimulus activation of response channels: a psychophysiological analysis. *J. Exp. Psychol. Hum. Percept. Perform.* 14 331–344. 10.1037/0096-1523.14.3.331 2971764

[B26] Green C. S. Bavelier D. (2003). Action video game modifies visual selective attention. *Nature* 423 534–537. 10.1038/nature01647 12774121

[B27] Green C. S. Bavelier D. (2007). Action-video-game experience alters the spatial resolution of vision. *Psychol. Sci.* 18 88–94. 10.1111/j.1467-9280.2007.01853.x 17362383 PMC2896830

[B28] Green C. S. Bavelier D. (2012). Learning, attentional control, and action video games. *Curr. Biol.* 22 R197–R206. 10.1016/j.cub.2012.02.012 22440805 PMC3461277

[B29] Green C. S. Pouget A. Bavelier D. (2010). Improved probabilistic inference as a general learning mechanism with action video games. *Curr. Biol.* 20 1573–1579. 10.1016/j.cub.2010.07.040 20833324 PMC2956114

[B30] Hackley S. A. Miller J. (1995). Response complexity and precue interval effects on the lateralized readiness potential. *Psychophysiology* 32 230–241. 10.1111/j.1469-8986.1995.tb02952.x 7784531

[B31] Ho T. Brown S. van Maanen L. Forstmann B. U. Wagenmakers E.-J. Serences J. T. (2012). The optimality of sensory processing during the speed-accuracy tradeoff. *J. Neurosci.* 32 7992–8003. 10.1523/jneurosci.0340-12.2012 22674274 PMC3388609

[B32] Kokkinakis A. V. Cowling P. I. Drachen A. Wade A. R. (2017). Exploring the relationship between video game expertise and fluid intelligence. *PLos One* 12:e0186621. 10.1371/journal.pone.0186621 29141019 PMC5687598

[B33] Kopp B. Wessel K. (2010). Event-related brain potentials and cognitive processes related to perceptual-motor information transmission. *Cogn. Affect. Behav. Neurosci.* 10 316–327. 10.3758/cabn.10.2.316 20498353

[B34] Kowalczyk N. Shi F. Magnuski M. Skorko M. Dobrowolski P. Kossowski B. (2018). Real-time strategy video game experience and structural connectivity - A diffusion tensor imaging study. *Hum. Brain Mapp.* 39 3742–3758. 10.1002/hbm.24208 29923660 PMC6866322

[B35] Li X. Huang L. Li B. Wang H. Han C. (2020). Time for a true display of skill: top players in League of Legends have better executive control. *Acta Psychol.* 204:103007. 10.1016/j.actpsy.2020.103007 32000064

[B36] Lindenberger U. Lövdén M. (2019). Brain plasticity in human lifespan development: the exploration–selection–refinement model. *Annu. Rev. Dev. Psychol.* 1 197–222. 10.1146/annurev-devpsych-121318-085229

[B37] Loevden M. Backman L. Lindenberger U. Schaefer S. Schmiedek F. (2010). A theoretical framework for the study of adult cognitive plasticity. *Psychol. Bull.* 136 659–676. 10.1037/a0020080 20565172

[B38] Lopez-Calderon J. Luck S. J. (2014). ERPLAB: an open-source toolbox for the analysis of event related potentials. *Front. Hum. Neurosci.* 8:213. 10.3389/fnhum.2014.00213 24782741 PMC3995046

[B39] Lu J. Li G. Gong D. Hu Q. (2014). Partial information transmission can be found in music attributes. *Neuroreport* 25 190–193. 10.1097/wnr.0000000000000104 24323126 PMC3906251

[B40] Luck S. J. (2014). *An Introduction to the Event-Related Potential Technique.* Cambridge, MA: MIT press.

[B41] Masaki H. Wild-Wall N. Sangals J. Sommer W. (2004). The functional locus of the lateralized readiness potential. *Psychophysiology* 41 220–230. 10.1111/j.1469-8986.2004.00150.x 15032987

[B42] Miller J. Hackley S. A. (1992). Electrophysiological evidence for temporal overlap among contingent mental processes. *J. Exp. Psychol. Gen.* 121 195–209. 10.1037/0096-3445.121.2.195 1318354

[B43] Osman A. Bashore T. R. Coles M. G. Donchin E. Meyer D. E. (1992). On the transmission of partial information: inferences from movement-related brain potentials. *J. Exp. Psychol. Hum. Percept. Perform.* 18 217–232. 10.1037/0096-1523.18.1.217 1532189

[B44] Qiu N. Ma W. Fan X. Zhang Y. Li Y. Yan Y. (2018). Rapid improvement in visual selective attention related to action video gaming experience. *Front. Hum. Neurosci.* 12:47. 10.3389/fnhum.2018.00047 29487514 PMC5816940

[B45] Richler J. J. Wong Y. K. Gauthier I. (2011). Perceptual expertise as a shift from strategic interference to automatic holistic processing. *Curr. Direct. Psychol. Sci.* 20 129–134. 10.1177/0963721411402472 21643512 PMC3104280

[B46] Saalmann Y. B. Pinsk M. A. Wang L. Li X. Kastner S. (2012). The pulvinar regulates information transmission between cortical areas based on attention demands. *Science* 337 753–756. 10.1126/science.1223082 22879517 PMC3714098

[B47] Smulders F. T. Kok A. Kenemans J. L. Bashore T. R. (1995). The temporal selectivity of additive factor effects on the reaction process revealed in ERP component latencies. *Acta Psychol.* 90 97–109. 10.1016/0001-6918(95)00032-p 8525879

[B48] Stelmach L. B. Herdman C. M. (1991). Directed attention and perception of temporal order. *J. Exp. Psychol. Hum. Percept. Perform.* 17 539–550. 10.1037/0096-1523.17.2.539 1830091

[B49] Sternberg S. (1969). The discovery of processing stages: extensions of donders’ method. *Acta Psychol.* 30 276–315. 10.1016/0001-6918(69)90055-9

[B50] Takacs A. Bluschke A. Kleimaker M. Munchau A. Beste C. (2020). Neurophysiological mechanisms underlying motor feature binding processes and representations. *Hum. Brain Mapp.* 42 1313–1327. 10.1002/hbm.25295 33236838 PMC7927300

[B51] Uccelli S. Palumbo L. Harrison N. R. Bruno N. (2020). Asymmetric effects of graspable distractor disks on motor preparation of successive grasps: a behavioural and event-related potential (ERP) study. *Int. J. Psychophysiol.* 158 318–330. 10.1016/j.ijpsycho.2020.10.007 33164874

[B52] Walk A. M. Raine L. B. Kramer A. F. Cohen N. J. Hillman C. H. Khan N. A. (2020). Adiposity is related to neuroelectric indices of motor response preparation in preadolescent children. *Int. J. Psychophysiol.* 147 176–183. 10.1016/j.ijpsycho.2019.10.014 31756405

[B53] Wenger E. Kuehn S. Verrel J. Martensson J. Bodammer N. C. Lindenberger U. (2017). Repeated Structural imaging reveals nonlinear progression of experience-dependent volume changes in human motor cortex. *Cerebr. Cortex* 27 2911–2925. 10.1093/cercor/bhw141 27226440

[B54] Xia M. Liu T. Bai W. Zheng X. Tian X. (2019). Information transmission in HPC-PFC network for spatial working memory in rat. *Behav. Brain Res.* 356 170–178. 10.1016/j.bbr.2018.08.024 30170031

[B55] Yao D. Z. (2001). A method to standardize a reference of scalp EEG recordings to a point at infinity. *Physiol. Meas.* 22 693–711. 10.1088/0967-3334/22/4/305 11761077

[B56] Yao Y. Cui R. Li Y. Zeng L. Jiang J. Qiu N. (2020). Action real-time strategy gaming experience related to enhanced capacity of visual working memory. *Front. Hum. Neurosci.* 14:333. 10.3389/fnhum.2020.00333 33110407 PMC7489035

[B57] Zhang W. Guo L. Liu D. Xu G. (2020). The dynamic properties of a brain network during working memory based on the algorithm of cross-frequency coupling. *Cogn. Neurodyn.* 14 215–228. 10.1007/s11571-019-09562-9 32226563 PMC7090136

[B58] Zhang Y.-X. Tang D.-L. Moore D. R. Amitay S. (2017). Supramodal enhancement of auditory perceptual and cognitive learning by video game playing. *Front. Psychol.* 8:1086. 10.3389/fpsyg.2017.01086 28701989 PMC5487488

